# OLGA: fast computation of generation probabilities of B- and T-cell receptor amino acid sequences and motifs

**DOI:** 10.1093/bioinformatics/btz035

**Published:** 2019-01-18

**Authors:** Zachary Sethna, Yuval Elhanati, Curtis G Callan, Aleksandra M Walczak, Thierry Mora

**Affiliations:** 1 Joseph Henry Laboratories, Princeton University, Princeton, NJ, USA; 2 Laboratoire de physique de l'Ecole normale supérieure (PSL University), Centre national de la recherche scientifique, Sorbonne University, University Paris-Diderot, Paris, France

## Abstract

**Motivation:**

High-throughput sequencing of large immune repertoires has enabled the development of methods to predict the probability of generation by V(D)J recombination of T- and B-cell receptors of any specific nucleotide sequence. These generation probabilities are very non-homogeneous, ranging over 20 orders of magnitude in real repertoires. Since the function of a receptor really depends on its protein sequence, it is important to be able to predict this probability of generation at the amino acid level. However, brute-force summation over all the nucleotide sequences with the correct amino acid translation is computationally intractable. The purpose of this paper is to present a solution to this problem.

**Results:**

We use dynamic programming to construct an efficient and flexible algorithm, called OLGA (Optimized Likelihood estimate of immunoGlobulin Amino-acid sequences), for calculating the probability of generating a given CDR3 amino acid sequence or motif, with or without V/J restriction, as a result of V(D)J recombination in B or T cells. We apply it to databases of epitope-specific T-cell receptors to evaluate the probability that a typical human subject will possess T cells responsive to specific disease-associated epitopes. The model prediction shows an excellent agreement with published data. We suggest that OLGA may be a useful tool to guide vaccine design.

**Availability and implementation:**

Source code is available at https://github.com/zsethna/OLGA.

**Supplementary information:**

Supplementary data are available at *Bioinformatics* online.

## 1 Introduction

The ability of the adaptive immune system to recognize foreign peptides, while avoiding self peptides, depends crucially on the specificity of receptor-antigen binding and the diversity of the receptor repertoire. Immune repertoire sequencing (Repseq) of B- and T-cell receptors (BCR and TCR) (Heather *et al.*, 2017; Lindau and Robins, 2017; Six *et al.*, 2013; Woodsworth *et al.*, 2013) offers an efficient experimental tool to probe the diversity of full repertoires in healthy individuals (Freeman *et al.*, 2009; Howie *et al.*, 2015; Mora and Walczak, 2018; Pogorelyy *et al.*, 2017; Robins *et al.*, 2009, 2010; Weinstein *et al.*, 2009), in cohorts with specific conditions (DeWitt *et al.*, 2018; Emerson *et al.*, 2017; Faham *et al.*, 2017; Horns *et al.*, 2017; Jiang *et al.*, 2013; Komech *et al.*, 2018; Pogorelyy *et al.*, 2018a; Vollmers *et al.*, 2013) and evaluate the response to specific fluorescent MHC-multimers (Dash *et al.*, 2017; Glanville *et al.*, 2017). Recent work has shown that responding clonotypes often form disjoint clusters of similar amino acid sequences, which has lead to the identification of responsive amino acid motifs (Dash *et al.*, 2017; Glanville *et al.*, 2017). In order for these techniques to have practical applications in therapy and vaccine design, one needs a fast and efficient algorithm to evaluate which specific amino acid sequences and sequence motifs are likely to be generated and found in repertoires. We present a solution to this problem in the form of an algorithm and computational tool, called OLGA, which implements an exact computation of the generation probability of any BCR or TCR sequence (nucleotide or amino acid), or motif.

BCR and TCR are stochastically generated by choosing a germline genetic template in each of several cassettes of alternates [V, (D), or J] and then splicing them together with random nucleotide deletions and insertions at the junctions. Given a generative model, one can define the generation probability of any nucleotide sequence as the sum of the probabilities of all the generative events that can produce that sequence (Elhanati *et al.*, 2015, 2016; Marcou *et al.*, 2018; Murugan *et al.*, 2012). However, computing the generation probability of amino acid sequences by summing over all consistent nucleotide sequences is impractical: because of codon degeneracy, the number of nucleotide sequences to be summed grows exponentially with sequence length. OLGA is powered by an efficient dynamic programming method to exactly sum over generative events and obtain net probabilities of amino acid sequences and motifs.

We validate our algorithm by comparing its results and performance to Monte-Carlo sampling estimates. We present results using publicly available data for both TCR *α* (TRA, Pogorelyy *et al.*, 2017) and *β* (TRB, Emerson *et al.*, 2017) chains and BCR heavy chains (IGH, DeWitt *et al.*, 2016) of humans, and TRB of mice (Sethna *et al.*, 2017). We applied OLGA to a TCR database that catalogs the different CDR3 amino acid sequences responding to a variety of different epitopes associated with disease (Shugay *et al.*, 2018). We computed the generation probability of particular CDR3 amino acid sequences, as well as the net generation probability of all the TCR that respond to a particular epitope. Finally, we discuss OLGA’s applications in vaccine design and other therapeutic contexts.

## 2 Materials and methods

### 2.1 Stochastic model of VDJ recombination

V(D)J recombination is a stochastic process involving several events (gene template selection, terminal deletions from the templates, random insertions at the junctions), each of which has a set of possible outcomes chosen according to a discrete probability distribution. The probability $P_{\text{gen}}^{\text{rec}}(E)$ of any generation event *E*, defined as a combination of the above-mentioned processes is, for the TRB locus:

(1) $$\begin{array}{cc} & P_{\text{gen}}^{\text{rec}}(E)=P_{V}(V)P_{\text{DJ}}(D,J)P_{\text{delV}}(d_{V}|V)P_{\text{delJ}}(d_{J}|J)\\ & \times P_{\text{delD}}(d_{D},d\prime_{D}|D)P_{\text{insVD}}(\ell_{\text{VD}})p_{0}(m_{1})\left[\prod_{i=2}^{\ell_{\mathrm{VD}}}S_{\text{VD}}(m_{i}|m_{i-1})\right]\\ & \times P_{\text{insDJ}}(\ell_{\text{DJ}})q_{0}(n_{\ell_{\mathrm{DJ}}})\left[\prod_{i=1}^{\ell_{\mathrm{DJ}}-1}S_{\text{DJ}}(n_{i}|n_{i+1})\right], \end{array}$$

where (*V*, *D*, *J*) identify the choices of gene templates, $\left(d_{V},d_{D},d\prime_{D},d_{J}\right)$ are the numbers of deletions at each end of the segments, and $\left(m_{1},\ldots ,m_{\ell_{\mathrm{VD}}}\right)$ and $\left(n_{1},\ldots ,n_{\ell_{\mathrm{DJ}}}\right)$ are the untemplated inserted nucleotide sequences at the VD and DJ junctions. These variables specify the recombination event *E*, and are drawn according to the probability distributions ($P_{V},\;P_{\text{DJ}},\;P_{\text{delV}},\;P_{\text{delD}},\;P_{\text{delJ}},\;P_{\text{insVD}},\;P_{\text{insDJ}}$, *p*_0_, *q*_0_, $S_{\text{VD}},\;S_{\text{DJ}}$). The inserted segments are drawn according to a Markov process starting with the nucleotide distribution *p*_0_ and with the transition matrix *R*, and running from the 5′ side (left to right) for the VD segment, and from the 3′ side (right to left) from the DJ segment. Similar models can be defined for the *α* chain or for BCR chains. Although here we describe the method for TRB only, it is also implemented for other chains in the software.

Since the same nucleotide sequence can be created by more than one specific recombination event, the generation probability of a nucleotide sequence is the sum of the probabilities of all possible events that generate the sequence: $P_{\text{gen}}^{\text{nt}}(\sigma )=\sum_{E\to \sigma }P_{\text{gen}}^{\text{rec}}(E),$ where the sum is over all recombination events *E* that produce the sequence $\sigma =(\sigma_{1},\ldots ,\sigma_{n})$. The probability of generation of an amino acid sequence, $a=(a_{1},\ldots ,a_{L})$ is the sum of the probabilities of all nucleotide sequences that translate into the amino acid sequence:

(2) $$P_{\text{gen}}^{\text{aa}}(a_{1},\ldots ,a_{L})=\sum_{\sigma ∼a}P_{\text{gen}}^{\text{nt}}(\sigma_{1},.,\sigma_{3L})=\sum_{E\to \sigma ∼a}P_{\text{gen}}^{\text{rec}}(E),$$

where the ∼ sign indicates that σ translates into a. We can generalize this approach to any scheme that groups nucleotide triplets, or codons, into arbitrary classes, which we still denote by σ∼a. In the formulation above, these classes simply group together codons with the same translation according to the standard genetic code. In an example of generalization, all codons that code for amino acids with a common chemical property, e.g. hydrophobicity or charge, could be grouped into a single class. In that formulation, $\left(a_{1},\ldots ,a_{L}\right)$ would correspond to a sequence of symbols denoting that property. More generally, any grouping of amino acids can be chosen (including one where any amino acid is acceptable), and the partition can be position dependent. Thus, the generation probability of arbitrary ‘motifs’ can be queried. In the following, for ease of exposition, we restrict our attention to the case where a is an amino acid sequence.

### 2.2 Dynamic programming computation of the generation probability of amino acid sequences

We now give an overview of how OLGA computes Eq. 2 without performing the sum explicitly, using dynamic programming. Supplementary Figures S1 and S2 give a graphical overview of the method, and details of the method implementation can be found in Supplementary Sections I and II and in the code manual. Given the genomic nucleotide sequences of the possible gene templates, together with a specific model of the type described in Eq. 1, the algorithm computes the net probability of generating a recombined gene with a given CDR3 amino acid sequence under a given set of V and J gene choices.

**Fig. 1.**
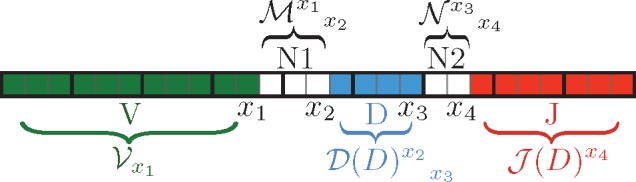
Partitioning a CDR3 sequence: boxes correspond to nucleotides and are indexed by integers. Each group of three boxes (identified by heavier boundary lines) corresponds to an amino acid. The nucleotide positions $x_{1},\ldots ,x_{4}$ identify the boundaries between different elements of the partition. The V, M, D(D), N and J(D) matrices define cumulated weights corresponding to each of the five elements

Each recombination event implies an annotation of the CDR3 sequence, assigning a different origin to each nucleotide (V, N1, D, N2, or J, where N1 and N2 are the VD and DJ insertion segments, respectively) that parses the sequence into five contiguous segments (see schematic in Fig. 1). The principle of the method is to sum over the probabilities of all choices of nucleotides consistent with the known amino acid sequence, over the possible locations of the four boundaries (*x*_1_, *x*_2_, *x*_3_ and *x*_4_) between the five segments, and over the possible V, D and J genomic templates (Fig. 1). We do this in a recursive way using matrix operations by defining weights that accumulate the probabilities of events from the left of a position *x* (i.e. up to *x*), and weights that accumulate events from the right of *x* (i.e. from *x *+* *1 on). Specifically, we define the following index notation: $X_{x}$ with a subscript called left index, accumulates weights from the left of *x*; $Y^{x}$, with a superscript called right index, accumulates weights from the right of *x*; a matrix X yx corresponds to accumulated weights from position *x *+* *1 to *y* (as will be explained shortly, these objects may have suppressed nucleotide indices as well). $P_{\text{gen}}^{\text{aa}}$ is calculated recursively by matrix-like multiplications as:

(3) $$P_{\text{gen}}^{\text{aa}}(a)=\sum_{x_{1},x_{2},x_{3},x_{4}}V_{x_{1}}M^{x_{1}}{}_{x_{2}}\sum_{D}\left[D{\left(D\right)}^{x_{2}}{}_{x_{3}}N^{x_{3}}{}_{x_{4}}J{\left(D\right)}^{x_{4}}\right].$$

The vector $V_{x}$ corresponds to a cumulated probability of the V segment finishing at position *x*; M yx is the probability of the VD insertion extending from *x *+* *1 to *y*; N yx is the same for DJ insertions; D yx corresponds to weights of the D segment extending from *x *+* *1 to *y*, conditioned on the D germline choice being *D*; $J^{x}(D)$ gives the weight of J segments starting at position *x *+* *1 conditioned on the D germline being *D*. This *D* dependency is necessary to account for the dependence between the D and J germline segment choices (Murugan *et al.*, 2012). All the defined vectors and matrices depend implicitly on the amino acid sequence $\left(a_{1},\ldots ,a_{L}\right)$, but we leave this dependency implicit to avoid making the notation too cumbersome.

Because we are dealing with amino acid sequences encoded by triplet nucleotide codons, we need to keep track of the identity of the nucleotide at the beginning or the end of a codon. Depending on the position of the index *x* in the codon, the objects defined above may be vectors of size four (or 4 × 4 matrices) in the suppressed nucleotide index. We use conventions that depend on whether we are considering left or right indices, as follows.

If *x* is a multiple of three, i.e. x=0 (mod 3), then we do not keep nucleotide information and both $X_{x}$ and $Y^{x}$ are scalars (whether *x* is a left or a right index). If x=1 (mod 3), then $X_{x}$ must be interpreted as a row vector of four numbers, $X_{x}(\sigma ),\;\sigma =A,T,G,C$, corresponding to the cumulated probability weight that the nucleotide at position *x* (first position of the codon) takes value *σ*. If x=2 (mod 3), then $X_{x}$ is also a row vector of four numbers, $X_{x}(\sigma )$, but with a different interpretation: it corresponds to the cumulated probability up to position *x*, with the additional constraint that the nucleotide at position *x *+* *1 (the last position in the codon) *can* take value *σ* (the value is 0 otherwise). For right indices, the interpretation is reversed and the entries are column vectors: when x=1 (mod 3) the $Y^{x}$ is a column vector containing the cumulated weights from *x *+* *1 onwards, with the constraint that the nucleotide at *x can* be *σ*, and when x=2 (mod 3), it is the probability weight that the nucleotide at position *x *+* *1 *is σ*. Generalizing to matrices, X yx is a 4 × 4, 4 × 1, 1 × 4, or 1 × 1 matrix depending on whether the *x* and *y* positions are multiples of 3 or not, with the same rules as for vectors for each type of index.

Entries with left indices are interpreted as row vectors, and entries with right indices as column vectors. Thus, in Eq. 3 contractions between left and right indices correspond to dot products over the four nucleotides when the index is not a multiple of three, and simply a product of scalars when it is.

The entries of the matrices corresponding to the germline segments, V, D(D) and J(D), can be calculated by simply summing over the probabilities of different germline nucleotide segments compatible with the amino acid sequence $\left(a_{1},\ldots ,a_{L}\right)$ with conditions on deletions to achieve the required segment length. For instance, the V matrix elements are given by:

(4) $$\begin{array}{c} V_{x}(\sigma )=\sum_{V}P_{V}(V)P_{\text{delV}}(l_{V}-x)I(s_{x}^{V}=\sigma )I(s_{1:x}^{V}∼a_{1:i})\;\text{if}\;u=1\\ V_{x}(\sigma )=\sum_{V}P_{V}(V)P_{\text{delV}}(l_{V}-x)I((s_{1:x}^{V},\sigma )∼a_{1:i})\;\text{if}\;u=2,\\ V_{x}=\sum_{V}P_{V}(V)P_{\text{delV}}(l_{V}-x)I(s_{1:x}^{V}∼a_{1:i})\;\text{if}\;u=3, \end{array}$$

where x=3(i−1)+u, i.e. *x* is the *u*th nucleotide of the *i*th codon, $s^{V}$ the sequence of the V germline gene, and I the indicator function. The ∼ sign is generalized to incomplete codons so that it returns a true value if there exists a codon completion that agrees with the motif a. Detailed formulas for the other segments are derived using the same principles and are given in the SI Appendix. The sums in Eq. 4 (and equivalent expressions for J) can be restricted to particular germline genes to compute the generation probability of particular VJ-CDR3 combinations.

The entries of the insertion segment N1 are calculated using the following formula:

(5) M yx=PinsVD(y−x)LaiuTai+1…Taj−1Rajv,

with y=3(j−1)+v (and x=3(i−1)+u as in Eq. 4). The transfer matrix

(6) $$T_{a}(\tau ,\sigma )=\sum_{(n_{1},n_{2},\sigma )∼a}S_{\text{VD}}(\sigma |n_{2})S_{\text{VD}}(n_{2}|n_{1})S_{\text{VD}}(n_{1}|\tau )$$

corresponds to the probability of inserting a codon coding for *a* and ending with nucleotide *σ*, knowing that the previous codon ended with nucleotide *τ*. $L_{a}^{u}$ and $R_{a}^{v}$ are vectors or matrices with different definitions depending on the values of *x* and *y* modulo 3, corresponding to the probabilities of inserting incomplete codons on the left and right ends of the insertion segment. Eq. 5 is only valid for *j *>* i*, but similar formulas describe the case *i *=* j*. The precise definitions of *L* and *R*, the *i *=* j* case, and the formulas for N and the N2 insertion segment, which is exactly equivalent, are all given in detail in the SI Appendix.

The matrix product of Eq. 5 can be calculated recursively, requiring only 4 × 4 matrix multiplications. Thus, all M yx elements can be calculated in $O(L^{2})$ operations, instead of the exponential time that would be required using brute-force summation over nucleotides in degenerate codons. Finally, since the sums of Eq. 3 can also be done recursively through *L *×* L* matrix operations, the whole procedure has $O(L^{2})$ computational complexity.

## 3 Results

### 3.1 Method validation

**Fig. 2.**
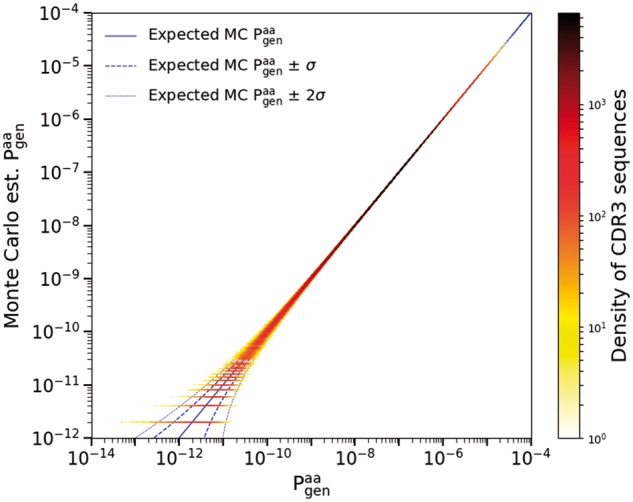
Monte Carlo estimate of the generation probability of amino acid CDR3 sequences, $P_{\text{gen}}^{\text{aa}}$, versus OLGA’s predictions (mouse TRB). The horizontal lines at the lower left of the plot represent CDR3s that were generated once, twice, etc., in the MC sample. The one- and two-sigma curves display the deviations from exact equality between simulated and computed $P_{\mathrm{gen}}$ to be expected on the basis of Poisson statistics

To verify the correctness of the OLGA code, we compared its predictions for generation probabilities to those estimated by Monte Carlo (MC) sequence generation (Pogorelyy *et al.*, 2018a). MC estimation is done by drawing events from a given generative model, binning according to the resulting CDR3 amino acid sequence, and normalizing by the total number of recombination events. The scatter plot of the estimated generation probabilities for these sequences against the values predicted by OLGA gives a direct test of the algorithm. As MC estimation is susceptible to Poisson sampling noise, it is important to ensure that enough events are drawn to accurately assess the generative probabilities of individual CDR3 sequences. For this reason, we made the comparison using a generative model inferred from a mouse, rather than human, T cell repertoire, because of the significantly lower entropy of mouse repertoires (Sethna *et al.*, 2017). The specific model was inferred by IGoR (Marcou *et al.*, 2018) using ∼70 000 out-of-frame TRB sequences from a mature mouse thymus. MC estimation was done by generating $5\times 10^{11}$ recombination events, from which the first 10^6^ unique CDR3 amino acid sequences are counted to serve as a sample for the comparison. This procedure provided good sequence coverage, with >98% of sequences generated at least twice and >95% of sequences generated at least 10 times. As Figure 2 shows for mouse TRB (see Supplementary Fig. S3 for human TRA), MC estimation and OLGA calculation are in agreement (up to Poisson noise in the MC estimate). The Kullback-Leibler divergence between the two distributions, a formal measure of their agreement, is a mere $4.82\times 10^{-7}$ bits.

### 3.2 Comparison of performance with existing methods

**Fig. 3.**
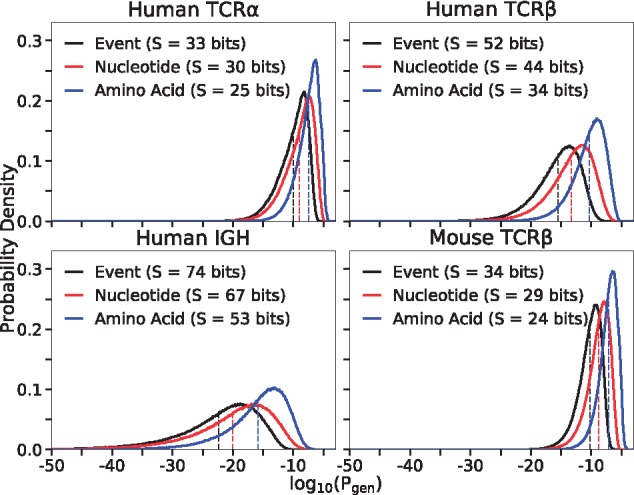
Distributions of probabilities of recombination events ($P_{\text{gen}}^{\text{rec}}$), nucleotide CDR3 sequences ($P_{\text{gen}}^{\text{nt}}$) and CDR3 amino acid sequences ($P_{\text{gen}}^{\text{aa}}$) in different contexts. Each curve is determined by Monte Carlo sampling of 10^6^ productive sequences for the indicated locus, and computing its generation probabilities at the three different levels. Entropies in bits (*S*) are, up to a ln(2)/ln(10) factor, the negative of the mean of each distributions, indicated by dotted lines

We compared the performance of OLGA to other methods. Direct calculation of amino acid sequence generation probability using OLGA is orders of magnitude faster than the two possible alternative methods: MC estimation (as described above), or exhaustive enumeration of the generative events giving rise to a given amino acid sequence. OLGA took six CPU hrs to compute the generation probabilities of the 10^6^ amino acid sequences, i.e. 47 seqs/CPU/sec for mouse TRB (see Supplementary Section III and Supplementary Table S1 for runtimes of other loci). By comparison, MC estimation required 4313 CPU hrs. The scaling for the MC estimation does not depend on the number of queried sequences, but instead is determined by the number of recombinations needed to control the Poisson noise, which scales inversely with generation probability. In practice, to determine the generation probability of a typical sequence (which can be as low $10^{-20}$, see Fig. 3 and below), one needs to generate very large datasets, and thus the generation probability of many sequences cannot be calculated by the MC method.

Alternatively, one could list all possible nucleotide sequences that translate to a particular amino acid CDR3 and sum the generation probabilities of each nucleotide sequence, using the IGoR algorithm (Marcou *et al.*, 2018). Each amino acid sequence in the mouse validation sample is, on average, coded for by 1.84 billion nucleotide sequences (and much more for human TRB). Since IGoR computes generation probabilities of nucleotide sequences at the rate of ∼60 seqs/CPU/sec, it would take ∼8500 CPU hrs to compute the generation probability of a *single* amino acid sequence. A systematic comparison of OLGA with IGoR (Supplementary Fig. S4) and MC estimation (Supplementary Figs S4 and S5) as a function of the number of analyzed sequences and their CDR3 lengths shows that OLGA is faster than both other methods for all practical purposes (see Supplementary Section IV for details).

### 3.3 Distribution of generation probabilities and diversity

V(D)J recombination produces very diverse repertoires of nucleotide sequences, with a very broad distribution of generation probabilities spanning up to 20 orders of magnitude (Elhanati *et al.*, 2015; Murugan *et al.*, 2012). This distribution gives a comprehensive picture of the diversity of the process, and can be used to recapitulate many classical diversity measures (Mora and Walczak, 2018), and to predict the overlap between the repertoires of different individuals (Elhanati *et al.*, 2018). In particular, the opposite of the mean logarithm of the generation probability, $-\langle {\;\text{log}\;}_{2}P_{\text{gen}}\rangle$, is equal to the entropy of the process. While previous work focused on nucleotide sequence generation, OLGA allows us to compute this distribution for amino acid sequences.

Figure 3 shows the distribution of $P_{\text{gen}}^{\text{aa}}$ for four loci: human and mouse TRB, human TRA and human IGH, and compares it to the distributions of nucleotide sequence generation probabilities, $P_{\text{gen}}^{\text{nt}}$, and recombination event probabilities, $P_{\text{gen}}^{\text{rec}}$. While all these datasets are based on DNA RepSeq, we checked that the generation probability distribution was robust to the choice of protocol by computing the TRB distribution for independent datasets generated by RNA RepSeq (Sims *et al.*, 2016; Wang *et al.*, 2010; Wu *et al.*, 2018) (Supplementary Figs S6 and S7, and Supplementary Section V). The generation models used here and elsewhere in this paper were taken from Marcou *et al.* (2018), except for the human TRB model which was relearned using IGoR from one individual in Emerson *et al.* (2017) as a check. Going from recombination events to nucleotide sequences to amino acid sequences leads to substantial shifts in the distribution, and corresponding drops in entropies, as the distribution is progressively coarse-grained. Higher generation probability of a given receptor sequence leads to higher chance of finding it in any given individual. Generation probabilities may be constrasted to the scale set by the inverse of the number of independent recombination events [estimated between 10^8^ (Qi *et al.*, 2014) and 10^10^ (Lythe *et al.*, 2016) for human TCR]. Generation probabilities above this limit ($10^{-10}$ to $10^{-8}$ for human TCR) can be considered ‘large’ as the corresponding receptor will almost surely exist in each individual (Elhanati *et al.*, 2018). Another relevant scale to distinguish small from large generation probabilities is given by their geometric mean (dashed lines in Fig. 3).

### 3.4 Cross-species generation probabilities

While distinct species differ in their generation mechanisms, they may yet be able to generate the same CDR3s. Using OLGA, we computed the probabilities of producing human TRB CDR3s by the mouse recombination model, and vice versa (details in Supplementary Section VI). An impressive 72.6% of human CDR3s can theoretically be produced by mice, and 100% of mouse CDR3s can be produced by humans. While cross-species generation probabilities are lower than intra-species ones (Supplementary Fig. S8), they are correlated (Supplementary Fig. S9). These results suggest that CDR3s observed in the repertoires of humanized mouse models of human diseases could be relevant for predicting their presence in human repertoires as well. OLGA allows for evaluating this potential, and could be used to inform clinical trials.

### 3.5 Generation probability of specific TCR

**Fig. 4.**
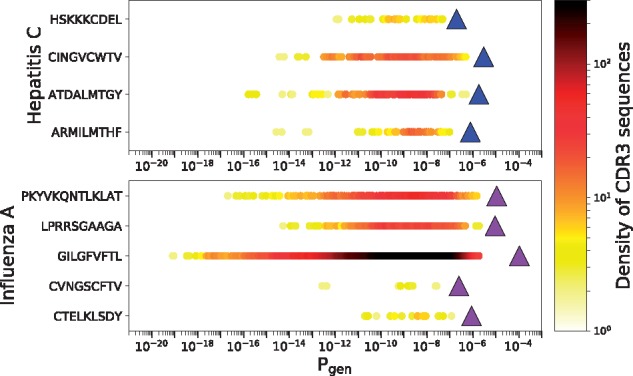
Generation probabilities of human CDR3s that respond to hepatitis C and influenza A epitopes. $P_{\text{gen}}^{\text{aa}}$ of sequences that respond to an epitope are plotted as circles (color encodes density of the points). The fraction of the repertoire specific to each epitope ($P_{\text{gen}}^{\text{func}}$ as defined in Eq. 7) is obtained as the sum of the $P_{\text{gen}}^{\text{aa}}$ for each of the corresponding sequences (values plotted as triangles) (Color version of this figure is available at *Bioinformatics* online.)

We can use OLGA to assess the total fraction of the generated repertoire that is specific to any given epitope, simply by summing the generation probabilities of all TRB sequences known to bind specifically to that epitope:

(7) $$P_{\text{gen}}^{\text{func}}(\text{epitope})=\sum_{a\;|\;\text{epitope}}P_{\text{gen}}^{\text{aa}}(a),$$

where ‘a|epitope’ means that the amino acid sequence a recognizes the epitope. Many experiments, based e.g. on multimer sorting assays (Dash *et al.*, 2017; Glanville *et al.*, 2017) or T-cell culture assays, have established lists of epitope-specific TCR sequences for a number of disease-related epitopes. We used the VDJdb database (Shugay *et al.*, 2018), which aggregates such experiments, to compute $P_{\text{gen}}^{\text{func}}$ of all TRB known to be reactive against several epitopes. In Figure 4 we show results for four epitopes associated with Hepatitis C, and five epitopes associated with Influenza A. The net fraction of the repertoire specific to these epitopes ($10^{-7}$ to $10^{-4}$) is large in the sense defined above, meaning that any individual is likely to have many copies of reactive T cells in their naive repertoire.

**Fig. 5.**
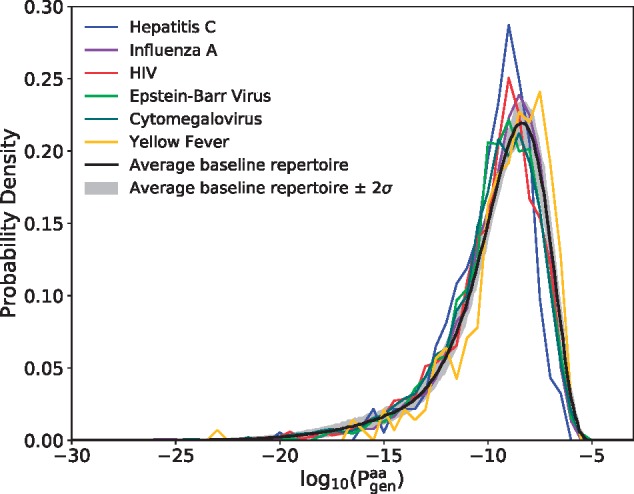
Distributions of TRB generation probabilities $P_{\text{gen}}^{\text{aa}}$ for sequences in the VDJdb database that bind to any epitopes of six different viruses (colored curves). For comparison, we plot (black curve) the same distribution for the unsorted TRB repertoire of a typical healthy subject; the 2σ variance represents biological variability across multiple individuals [data from Emerson *et al.* (2017)] (Color version of this figure is available at *Bioinformatics* online.)

The presence of any specific TCR in the repertoire will be affected by the recombination probability of both its *α* and *β* chains, and also by function-dependent selective pressures. Assessing accurately the fraction of reactive TCRs in the blood is beyond the scope of this method. However, it is still interesting to ask whether epitope-specific TRB sequences had higher generation probabilities than regular sequences, either because of observational biases, or because the immune system might have evolved to make them more likely to be produced. To answer that question, we display in Figure 5 the $P_{\text{gen}}^{\text{aa}}$ distribution of the sequences listed in VDJdb that are specific to any epitope of each of six commonly studied viruses. For comparison we plot the $P_{\text{gen}}^{\text{aa}}$ distribution of the full TRB sequence repertoire of a healthy donor [data taken from Emerson *et al.* (2017)].

The viral distributions are very similar to each other, and also to the healthy repertoire background, meaning that the ability of a CDR3 to respond to a particular disease epitope is not strongly correlated with its generation probability. To see whether this result was confirmed in the case of a real infection, we repeated the same analysis on TRB RepSeq data from T-cells responding to three different types of pathogens (fungus, bacteria and toxin) (Becattini *et al.*, 2015). Consistently, we found that their distribution of generation probability was identical to that of naive sequences (Supplementary Fig. S10 and Supplementary Section VII).

### 3.6 Model accurately predicts the frequencies of sequences and of groups of specific sequences

**Fig. 6.**
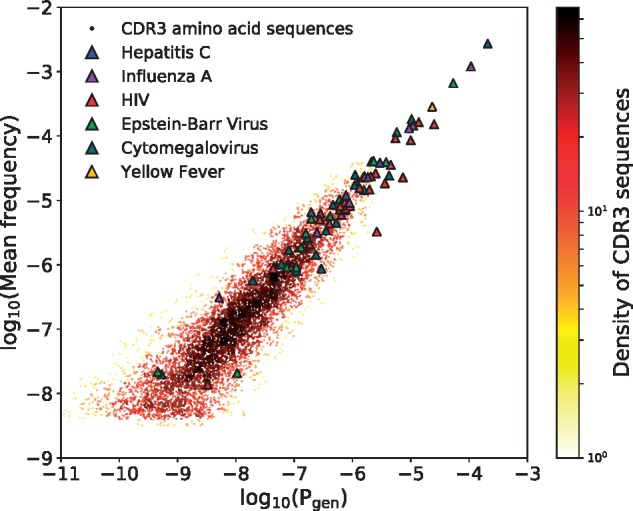
Mean occurrence frequencies across a collection of 658 human samples of all CDR3 sequences in the VDJdb database, plotted against their computed $P_{\text{gen}}^{\text{aa}}$ (dots, colored by their density in the plot). Also, the net occurrence frequency in the VDJdb database of epitope-related collections of sequences, plotted against their computed $P_{\text{gen}}^{\text{func}}$ (triangles, colored to identify the virus the epitope belongs to) (Color version of this figure is available at *Bioinformatics* online.)

To compare OLGA’s predictions with sequence occurrence frequencies in real data, we used the aggregated TRB repertoire of 658 human subjects described in Emerson *et al.* (2017) as a test resource. More specifically, we measured the frequencies in this large dataset of the specific CDR3 sequences contained in the VDJdb database (Shugay *et al.*, 2018), and compared them to the values assigned by OLGA. When measuring frequencies we discarded read count information, recording only the presence or absence of nucleotide sequences in each individual in order to eliminate effects of clonal expansion and PCR amplification bias, averaging over the 648 individuals in the Emerson *et al.* (2017) dataset to get reliable estimates of frequencies. Each sequence in the VDJdb database is displayed as a dot in Figure 6, and the resulting distribution shows a strong correspondence between mean frequency in the large dataset and the predicted $P_{\text{gen}}^{\text{aa}}$ of that sequence.

We then measured the fraction of CDR3s in the aggregated repertoire that is specific to epitopes associated with six viruses (using lists of specific sequences in VDJdb), and compared it to OLGA’s prediction, $P_{\text{gen}}^{\text{func}}$. The agreement was again excellent (triangles in Fig. 6). Again we observe that most epitope-specific sequence groups have large enough frequencies to be found in any individual. Thus, the model can be used to predict the size of repertoire subsets specific to any epitope, as long as specificity data are available for this epitope.

### 3.7 Generation probability of sequence motifs

OLGA can also compute the generation probability of any sequence motif, encoded by a string of multiple choices of amino acids. We apply this feature to calculate the net frequency of epitope-specific motifs, and of motifs that define the TRA sequence of invariant T-cells.

T-cell sequences that can bind a given epitope are often closely related to each other, and this similarity can sometimes be partially captured by sequence motifs. We evaluated the probabilities of motifs derived from a recent study of CDR3 sequence specificity to a variety of epitopes (Dash *et al.*, 2017). We took two motifs corresponding to TRA and TRB VJ-CDR3 combinations of TCRs that are known to bind the Epstein-Barr virus HLA-A*0201-BMLF_1280_ (BMLF) and the influenza virus HLA-A*0201-M_158_ (M1) epitopes. The motifs and generation probabilities are reported in Table 1.

**Table 1.** Epitope-specific TCR motifs for the Epstein-Barr virus HLA-A*0201-BMLF_1280_ (BMLF) and influenza virus HLA-A*0201-M_158_ (M1) epitopes from Dash *et al.* (2017), and their generation probabilities

epitope: chain: V/J	CDR3 motif	$P_{\text{gen}}$
BMLF: *α*: 5/31	CAXD[NSDA]NARLMF	$1.8\cdot 10^{-7}$
BMLF: *β*: 20-1/1-2, 1-3	CSARDX[TV]GNX{0,}	$5.1\cdot 10^{-7}$
M1: *α*: 27/42	CAXGGSQGNLIF	$2.2\cdot 10^{-5}$
M1: *β*: 19/all	CASSXR[SA][STAG]X[ET]Q[YF]F	$1.7\cdot 10^{-6}$

*Note*: Each motif was associated with specific V/J gene choices. In the motifs we use the conventions: X, any one amino acid; [A.B], any one of the listed amino acids; X{0,}, arbitrary amino acid string.

As a second application, we estimated the probabilities of generating a TRA chain corresponding to one of the motifs associated with Mucosal associated invariant T cells (MAIT) and invariant natural killer T cells (iNKT). The motifs, which were collected from Gherardin *et al.* (2016), and their probabilities are shown in Table 2. The relatively high values for these motifs imply that these invariant chains are generated with high frequency in the primary repertoire and shared by all individuals, confirming the conclusions of Venturi *et al.* (2013).

**Table 2.** Generation probabilities of motifs corresponding to invariant T cell (iNKT and MAIT cells) TRA chain, assembled from sequence in Gherardin *et al.* (2016)

Type	V/J	CDR3 motif	$P_{\mathrm{gen}}$
iNKT	10/18	CVVSDRGSTLGRLYF	$1.26\cdot 10^{-6}$
MAIT	1-2/33	CAV[KSM]DSNYQLI[WF]	$1.79\cdot 10^{-5}$
MAIT	1-2/12	CAVMDSSYKLIF	$4.71\cdot 10^{-6}$
MAIT	1-2/20	CAVSDNDYKLSF	$3.11\cdot 10^{-7}$

## 4 Discussion

Because the composition of the immune repertoire results from a stochastic process, the frequency with which distinct T- and B-cell receptors are generated is a quantity of primary interest. This frequency is computationally difficult to evaluate because each amino acid sequence can be created by a very large number of recombination events. Our tool overcomes that challenge with dynamic programming, allowing it to process ∼50 sequences per second on a single CPU. In its current state OLGA can compute the probabilities of CDR3 sequences and motifs, with or without V/J restriction, of four chain loci (human and mouse TRB, human TRA and human IGH), but the list can readily be expanded by learning recombination models for other loci and species using IGoR (Marcou *et al.*, 2018) which shares the same model format. Obvious additions include the light chains of BCR (Toledano *et al.*, 2018), and more mouse models. While the algorithm evaluates the probability of single chains, recent analyses show that chain pairing in TCR is close to independent (Dupic *et al.*, 2018; Grigaityte *et al.*, 2017). The probability of generating a whole TCR receptor can thus be computed by taking the product over the two chains.

OLGA can be used to compute baseline receptor frequencies and to identify outlying sequences in repertoire sequencing datasets. In Elhanati *et al.* (2018) we used it to shed light on the question of public repertoires—composed of sequences shared by many individuals—and predict quantitatively its origin by convergent recombination (Madi *et al.*, 2014, 2017; Venturi *et al.*, 2008). Deviations from the baseline expectancy have been used to identify disease-associated TCR from cohorts of patients (Emerson *et al.*, 2017; Faham *et al.*, 2017; Fuchs *et al.*, 2017; Seay *et al.*, 2016; Zhao *et al.*, 2016), and to identify clusters of reactive TCRs from tetramer experiments (Glanville *et al.*, 2017) and vaccination studies (Pogorelyy *et al.*, 2018b). Such estimates could be made faster and more reliable by OLGA, especially for rare sequences, and without the need for a negative control cohort (Pogorelyy *et al.*, 2018a). In the future, OLGA could be useful in vaccine and therapy design by focusing attention on clonotypes that are likely to be present in every individual.

We applied OLGA to an experimental database of TCR responding to a variety of disease-associated epitopes. These selected TCR do not differ in their generation probabilities from those of random TCR found in the blood of healthy donors. However, some viral epitopes bind a much larger fraction of the repertoire than others. This observation has potentially important consequences for vaccine design. Since vaccine epitopes stimulate TCR in a pre-existing repertoire, epitopes targeting receptor sequences that are more likely to be generated will have a higher success rate in a wider range of individuals. OLGA can be used to identify such epitopes by computing their specific repertoire fractions, $P_{\text{gen}}^{\text{func}}$. While our examples are restricted to TCR, OLGA can also handle BCR and could be used to compute the generation probabilities of BCR precursors of highly reactive or broadly neutralizing antibodies, and thus guide vaccine design in that case as well. The algorithm does not yet handle hypermutations, and extending it to include them would be a useful development.

## Supplementary Material

btz035_Supplementary_Information
