# Supplementary material for: OLGA: fast computation of generation probabilities of B- and T-cell receptor amino acid sequences and motifs
Source: Bioinformatics. 2019 Jan 18;35(17):2974–81. doi: 10.1093/bioinformatics/btz035 (PMC6735909; doi:10.1093/bioinformatics/btz035)
Supplement: btz035_Supplementary_Information [file btz035_supplementary_information.pdf]

Supplementary information for
OLGA: fast computation of generation probabilities of B- and T-cell receptor amino acid sequences and motifs

Zachary Sethna, Yuval Elhanati, Curtis G. Callan Jr., Aleksandra M. Walczak, Thierry Mora

I. ADDITIONAL MATRIX DEFINITIONS FOR VDJ ALGORITHM

Recall that the generative VDJ model is defined as:

$$P^{\rm rec}_{\rm gen}(E) = P_{\rm V}(V)P_{\rm DJ}(D,J)P_{\rm delV}(d_V|V)P_{\rm delJ}(d_J|J)P_{\rm delD}(d_D,d'_D|D)P_{\rm insVJ}(\ell_{\rm VD})p_0(m_1)\left[\prod_{i=2}^{\ell_{VD}} S_{\rm VD}(m_i|m_{i-1})\right] \times P_{\rm insDJ}(\ell_{\rm DJ})q_0(n_{\ell_{DJ}})\left[\prod_{i=1}^{\ell_{DJ}-1} S_{\rm DJ}(n_i|n_{i+1})\right], \quad (1)$$

with

$$P^{\rm aa}_{\rm gen}(a_1,\ldots,a_L) = \sum_{\boldsymbol{\sigma}\sim\boldsymbol{a}} P^{\rm nt}_{\rm gen}(\sigma_1,\ldots,\sigma_{3L}) = \sum_{E\to\boldsymbol{\sigma}\sim\mathbf{a}} P^{\rm rec}_{\rm gen}(E). \quad (2)$$

As described in the main text, the dynamic programming algorithm can be summarized by the summation over the positions x_1, x_2, x_3, and x_4 of the following matrix multiplication:

$$P^{\rm aa}_{\rm gen}(a_1,\ldots,a_L) = \sum_{x_1,x_2,x_3,x_4} \mathcal{V}_{x_1}\mathcal{M}^{x_1}{}_{x_2} \times \sum_D \left[\mathcal{D}(D)^{x_2}{}_{x_3}\mathcal{N}^{x_3}{}_{x_4}\mathcal{J}(D)^{x_4}\right]. \quad (3)$$

The interpretation of the left (subscript) and right (superscript) indices are detailed in the main text, and schematized in Fig. S1. The sums are performed iteratively using matrix multiplications, as detailed in Fig. ??. As in the main text, the nucleotide indices will often be suppressed along with the implicit dependence on the amino acid sequence $(a_1,\ldots,a_L)$. For a given nucleotide position x_j, it will be convenient to refer to the amino acid index, and the position in the codon (from both the left and the right), so we introduce the following (graphically shown in the cartoon below): $x_j = 3(i_j-1)+u_j$, and u, so that i_j encodes the codon that index x_j belongs to, and u_j its position

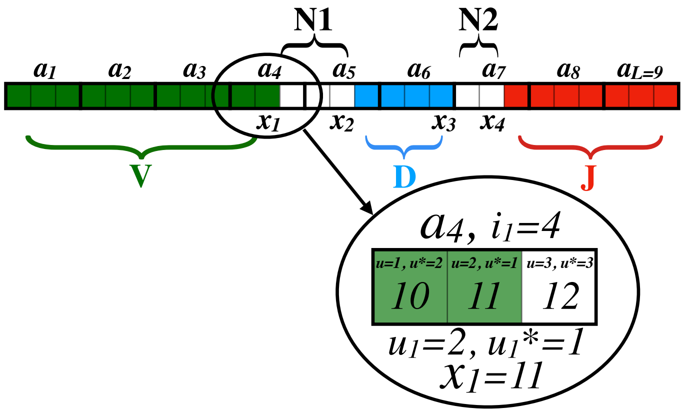

FIG. S1: Schematic of the partitioning of an amino acid sequence into sections for the purpose of constructing the probability matrices underlying the dynamic programming method for computing its net generation probability. The indexing conventions are also highlighted.

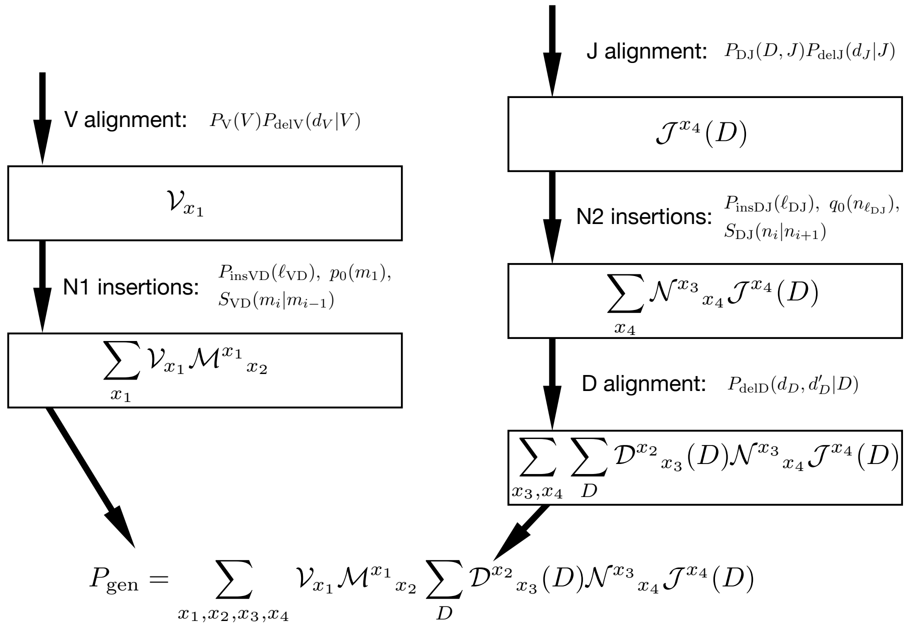

FIG. S2: Schematic of the OLGA VDJ algorithm implementation breakdown. Each of the 5 segments (V, N1, D, N2, J), and their associated model contributions, are considered from the edges of the CDR3 towards the inside. This is done both from the left side (V, N1) and the right side (D, N2, J) of the read to efficiently account for the correlations for the D and J genes. Including inner segments (N2, D, N2) requires summing over an index, indicating that all possible allowed start and end positions of the segment are considered.

(from 1 to 3) within that codon, while u_j^* denotes the position taken from the right of index x_j+1 within its codon, so that $u_j^*=2$ if $u_j=1$, $u_j^*=1$ if $u_j=2$, and $u_j^*=3$ if $u_j=3$.

We now define the explicit forms for each of the matrices (note that we retain the indexing x_j from Eq 3):

1. $\mathcal{V}_{x_1}$

Contribution from the templated V genes. $\mathcal{V}_{x_1}$ can be a 1x1 or 1x4 matrix depending on u_1. $\mathbf{s}^V$ is the sequence of the V germline gene (read $5'$ to $3'$) from the conserved residue (generally the cysteine C) to the end of the gene. l_V is the length of $\mathbf{s}^V$. These equations are given in the main text.

$$
\begin{aligned}
\mathcal{V}_{x_1}(\sigma) &= \sum_V P_{\rm V}(V)P_{\rm delV}(l_V-x_1|V)\mathbb{I}(s^V_{x_1}=\sigma)\mathbb{I}(\mathbf{s}^V_{1:x_1}\sim\mathbf{a}_{1:i_1}) \quad \text{if } u_1=1,\\
\mathcal{V}_{x_1}(\sigma) &= \sum_V P_{\rm V}(V)P_{\rm delV}(l_V-x_1|V)\mathbb{I}((\mathbf{s}^V_{1:x_1},\sigma)\sim\mathbf{a}_{1:i_1}) \quad \text{if } u_1=2,\\
\mathcal{V}_{x_1} &= \sum_V P_{\rm V}(V)P_{\rm delV}(l_V-x_1|V)\mathbb{I}(\mathbf{s}^V_{1:x_1}\sim\mathbf{a}_{1:i_1}) \quad \text{if } u_1=3.
\end{aligned}
\tag{4}
$$

2. $\mathcal{M}^{x_1}{}_{x_2}$

Contribution from the non-templated N1 insertions (VD junction). $\mathcal{M}^{x_1}{}_{x_2}$ is defined as the product of transfer matrices, and can be a 1x1, 1x4, 4x1, or 4x4 matrix depending on u_1 and u_2. The transfer matrices are defined by the summed contributions of the Markov insertion model of all codons consistent with the amino acid a (thus summations

are over nucleotides y, y_1, and y_2 to consider all allowed codons):

$$T_a(\tau,\sigma) = \sum_{(y_1,y_2,\sigma)\sim a} S_{\mathrm{VD}}(\sigma|y_2) S_{\mathrm{VD}}(y_2|y_1) S_{\mathrm{VD}}(y_1|\tau) \tag{5}$$

$$F_a(\tau,\sigma) = S_{\mathrm{VD}}(\sigma|\tau)\mathbb{I}[\exists\sigma',\sigma'' \text{ s.t. } (\sigma,\sigma',\sigma'')\sim a] \tag{6}$$

$$D_a(\tau,\sigma) = \sum_{(y_1,y_2,\sigma)\sim a} S_{\mathrm{VD}}(y_2|y_1) S_{\mathrm{VD}}(y_1|\tau) \tag{7}$$

$$lT_a(\tau,\sigma) = \sum_{(\tau,y,\sigma)\sim a} S_{\mathrm{VD}}(\tau|y) p_0(y) \tag{8}$$

$$lD_a(\tau,\sigma) = \sum_{(\tau,y,\sigma)\sim a} p_0(y) \tag{9}$$

If $i_2 > i_1$:

$$\mathcal{M}^{x_1}{}_{x_2} = P_{\mathrm{insVD}}(x_2 - x_1) L^{u_1}_{a_{i_1}} T_{a_{i_1+1}} \dots T_{a_{i_2-1}} R^{u_2}_{a_{i_2}} \tag{10}$$

where:

$$L^{u_1}_{a_{i_1}} = \begin{cases} lT_{a_{i_1}} & \text{if } u_1 = 1 \\ diag(p_0) & \text{if } u_1 = 2 \\ S_{\mathrm{VD}}^{-1} p_0 & \text{if } u_1 = 3 \end{cases} \quad \text{and} \quad R^{u_2}_{a_{i_2}} = \begin{cases} F_{a_{i_2}} & \text{if } u_2 = 1 \\ D_{a_{i_2}} & \text{if } u_2 = 2 \\ T_{a_{i_2}} \vec{1} & \text{if } u_2 = 3 \end{cases} \tag{11}$$

If $i_1 = i_2$:

$$\mathcal{M}^{x_1}{}_{x_2} = P_{\mathrm{insVD}}(x_2 - x_1) \times \begin{array}{c|ccc} & u_2 = 1 & u_2 = 2 & u_2 = 3 \\ \hline u_1 = 1 & \mathbb{1} & 0 & 0 \\ u_1 = 2 & lD_{a_{i_1}} & \mathbb{1} & 0 \\ u_1 = 3 & lT_{a_{i_1}}\vec{1} & diag(p_0)\vec{1} & 1 \end{array} \tag{12}$$

3. $\mathcal{D}(D)^{x_2}{}_{x_3}$

Contribution from the templated D genes. $\mathcal{D}(D)^{x_2}{}_{x_3}$ can be a 1x1, 1x4, 4x1, or 4x4 matrix depending on u_2^* and u_3^*. $\mathbf{s}^D$ is the sequence of the D germline gene (read $5'$ to $3'$) with length l_D.

$$\begin{aligned} \mathcal{D}(D)^{x_2}{}_{x_3}(\tau,\sigma) &= \sum_{d'_D} P_{\mathrm{delD}}(d_D, d'_D|D)\mathbb{I}[s^D_{d_D+1} = \tau]\mathbb{I}[s^D_{l_D-d'_D} = \sigma]\mathbb{I}[\mathbf{s}^D_{d_D+1:l_D-d'_D} \sim \mathbf{a}_{i_2:i_3}] \quad \text{if } u_2^* = 1 \text{ and } u_3^* = 1, \\ \mathcal{D}(D)^{x_2}{}_{x_3}(\tau,\sigma) &= \sum_{d'_D} P_{\mathrm{delD}}(d_D, d'_D|D)\mathbb{I}[s^D_{dD+1} = \tau]\mathbb{I}[(\mathbf{s}^D_{d_D+1:l_D-d'_D},\sigma) \sim \mathbf{a}_{i_2:i_3}] \quad \text{if } u_2^* = 1 \text{ and } u_3^* = 2, \\ \mathcal{D}(D)^{x_2}{}_{x_3}(\tau) &= \sum_{d'_D} P_{\mathrm{delD}}(d_D, d'_D|D)\mathbb{I}[s^D_{d_D+1} = \tau]\mathbb{I}[\mathbf{s}^D_{d_D+1:l_D-d'_D} \sim \mathbf{a}_{i_2:i_3}] \quad \text{if } u_2^* = 1 \text{ and } u_3^* = 3, \end{aligned} \tag{13}$$

$$\begin{aligned} \mathcal{D}(D)^{x_2}{}_{x_3}(\tau,\sigma) &= \sum_{d'_D} P_{\mathrm{delD}}(d_D, d'_D|D)\mathbb{I}[s^D_{l_D-d'_D} = \sigma]\mathbb{I}[(\tau,\mathbf{s}^D_{d_D+1:l_D-d'_D}) \sim \mathbf{a}_{i_2:i_3}] \quad \text{if } u_2^* = 2 \text{ and } u_3^* = 1, \\ \mathcal{D}(D)^{x_2}{}_{x_3}(\tau,\sigma) &= \sum_{d'_D} P_{\mathrm{delD}}(d_D, d'_D|D)\mathbb{I}[(\tau,\mathbf{s}^D_{d_D+1:l_D-d'_D},\sigma) \sim \mathbf{a}_{i_2:i_3}] \quad \text{if } u_2^* = 2 \text{ and } u_3^* = 2, \\ \mathcal{D}(D)^{x_2}{}_{x_3}(\tau) &= \sum_{d'_D} P_{\mathrm{delD}}(d_D, d'_D|D)\mathbb{I}[(\tau,\mathbf{s}^D_{d_D+1:l_D-d'_D}) \sim \mathbf{a}_{i_2:i_3}] \quad \text{if } u_2^* = 2 \text{ and } u_3^* = 3, \end{aligned} \tag{14}$$

$$\mathcal{D}(D)^{x_2}{}_{x_3}(\sigma) = \sum_{d'_D} P_{\text{delD}}(d_D, d'_D|D)\mathbb{I}[s^D_{l_D-d'_D} = \sigma]\mathbb{I}[\mathbf{s}^D_{d_D+1:l_D-d'_D} \sim \mathbf{a}_{i_2:i_3}] \quad \text{if } u^*_2 = 3 \text{ and } u^*_3 = 1,$$
$$\mathcal{D}(D)^{x_2}{}_{x_3}(\sigma) = \sum_{d'_D} P_{\text{delD}}(d_D, d'_D|D)\mathbb{I}[(\mathbf{s}^D_{d_D+1:l_D-d'_D}, \sigma) \sim \mathbf{a}_{i_2:i_3}] \quad \text{if } u^*_2 = 3 \text{ and } u^*_3 = 2, \tag{15}$$
$$\mathcal{D}(D)^{x_2}{}_{x_3} = \sum_{d'_D} P_{\text{delD}}(d_D, d'_D|D)\mathbb{I}[\mathbf{s}^D_{d_D+1:l_D-d'_D} \sim \mathbf{a}_{i_2:i_3}] \quad \text{if } u^*_2 = 3 \text{ and } u^*_3 = 3$$

where $d_D = l_D - (x_3 - x_2) - d'_D$

4. $\mathcal{N}^{x_3}{}_{x_4}$

Contribution from the non-templated N2 insertions (DJ junction). $\mathcal{N}^{x_3}{}_{x_4}$ is defined as the product of transfer matrices, and can be a 1x1, 1x4, 4x1, or 4x4 matrix depending on u^*_3 and u^*_4. The transfer matrices are defined by the summed contributions of the Markov insertion model of all codons consistent with the amino acid a (thus summations are over nucleotides y, y_1, and y_2 to consider all allowed codons):

$$T'_a(\tau, \sigma) = \sum_{(\sigma, y_2, y_1) \sim a} S_{\text{DJ}}(\sigma|y_2) S_{\text{DJ}}(y_2|y_1) S_{\text{DJ}}(y_1|\tau) \tag{16}$$
$$F'_a(\tau, \sigma) = S_{\text{DJ}}(\sigma|\tau)\mathbb{I}[\exists \sigma', \sigma'' \text{ s.t. } (\sigma'', \sigma', \sigma) \sim a] \tag{17}$$
$$D'_a(\tau, \sigma) = \sum_{(\sigma, y_2, y_1) \sim a} S_{\text{DJ}}(y_2|y_1) S_{\text{DJ}}(y_1|\tau) \tag{18}$$
$$lT'_a(\tau, \sigma) = \sum_{(\sigma, y, \tau) \sim a} S_{\text{DJ}}(\tau|y) q_0(y) \tag{19}$$
$$lD'_a(\tau, \sigma) = \sum_{(\sigma, y, \tau) \sim a} q_0(y) \tag{20}$$

If $i_4 > i_3$:

$$\mathcal{N}^{x_3}{}_{x_4} = P_{\text{insDJ}}(x_4 - x_3) {L'}^{u^*_3}_{a_{i_3}} T'_{a_{i_3+1}} \dots T'_{a_{i_4-1}} {R'}^{u^*_4}_{a_{i_4}} \tag{21}$$

where:

$${L'}^{u^*_3}_{a_{i_3}} = \begin{cases} F'_{a_{i_3}} & \text{if } u^*_3 = 1 \\ D'_{a_{i_3}} & \text{if } u^*_3 = 2 \\ T'_{a_{i_3}} \vec{1} & \text{if } u^*_3 = 3 \end{cases} \quad \text{and} \quad {R'}^{u^*_4}_{a_{i_4}} = \begin{cases} lT'_{a_{i_4}} & \text{if } u^*_4 = 1 \\ diag(q_0) & \text{if } u^*_4 = 2 \\ S^{-1}_{\text{DJ}} q_0 & \text{if } u^*_4 = 3 \end{cases} \tag{22}$$

If $i_3 = i_4$:

$$\mathcal{N}^{x_3}{}_{x_4} = P_{\text{insDJ}}(x_4 - x_3) \times \begin{array}{c|ccc} & u^*_4 = 1 & u^*_4 = 2 & u^*_4 = 3 \\ \hline u^*_3 = 1 & \mathbb{1} & lD'_{a_{i_3}} & lT'_{a_{i_3}} \vec{1} \\ u^*_3 = 2 & 0 & \mathbb{1} & diag(q_0)\vec{1} \\ u^*_3 = 3 & 0 & 0 & 1 \end{array} \tag{23}$$

5. $\mathcal{J}(D)^{x_4}$

Contribution from the templated J genes. $\mathcal{J}(D)^{x_4}$ can be a 1x1 or 4x1 matrix depending on u^*_4. $\mathbf{s}^J$ is the sequence of the J germline gene (read $5'$ to $3'$) and l_J gives the length of the sequence up to the conserved residue (generally

either F or W).

$$\begin{aligned}\mathcal{J}(D)^{x_4}(\tau) &= \sum_J P_{\mathrm{D,J}}(DJ)P_{\mathrm{delJ}}(d_J|J)\mathbb{I}(s^J_{d_J+1}=\tau)\mathbb{I}(\mathbf{s}^J_{d_J+1:l_J}\sim\mathbf{a}_{i_4:L}) \quad \text{if } u_4^*=1,\\ \mathcal{J}(D)^{x_4}(\tau) &= \sum_J P_{\mathrm{D,J}}(DJ)P_{\mathrm{delJ}}(d_J|J)\mathbb{I}((\tau,\mathbf{s}^J_{d_J+1:l_J})\sim\mathbf{a}_{i_4:L}) \quad \text{if } u_4^*=2,\\ \mathcal{J}(D)^{x_4} &= \sum_J P_{\mathrm{DJ}}(D,J)P_{\mathrm{delJ}}(d_J|J)\mathbb{I}(\mathbf{s}^J_{d_J+1:l_J}\sim\mathbf{a}_{i_4:L}) \quad \text{if } u_4^*=3.\end{aligned} \tag{24}$$

where $dJ = l_J - 3L - x_4 - 1$

II. VJ RECOMBINATION

The model used for VJ recombination is quite similar to the model for VDJ recombination with the main differences being the lack of a D segment and an N2 insertion segment. However, a strong correlation between V and J templates is observed in the TRA chain, so we include a joint V, J distribution to allow for this correlation. Due to this similarity, the algorithm used to compute $\mathrm{P_{gen}}$ is very similar. The VJ generative model is:

$$P^{\mathrm{rec}}_{\mathrm{gen}}(E) = P_{\mathrm{VJ}}(V,J)P_{\mathrm{delV}}(d_V|V)P_{\mathrm{delJ}}(d_J|J)\times P_{\mathrm{insVJ}}(\ell_{\mathrm{VJ}})p_0(m_1)\left[\prod_{i=2}^{\ell_{VJ}} S_{\mathrm{VJ}}(m_i|m_{i-1})\right] \tag{25}$$

with nucleotide and amino acid P_{gen}s being defined the same as for the VDJ recombination model (Eq 2). The dynamic programing algorithm also has a similar form to Eq 3, and can be summarized as (retaining all notation conventions from before):

$$P_{\mathrm{gen}}(a_1,\ldots,a_L) = \sum_{x_1,x_2}\sum_J \mathcal{V}(J)_{x_1}\mathcal{M}^{x_1}{}_{x_2}\mathcal{J}(J)^{x_2} \tag{26}$$

1. $\mathcal{V}(\mathcal{J})_{x_1}$

Contribution from the templated V genes.

$$\begin{aligned}\mathcal{V}(J)_{x_1}(\sigma) &= \sum_V P_{\mathrm{VJ}}(V,J)P_{\mathrm{delV}}(l_V-x_1|V)\mathbb{I}(s^V_{x_1}=\sigma)\mathbb{I}(\mathbf{s}^V_{1:x_1}\sim\mathbf{a}_{1:i_1}) \quad \text{if } u_1=1,\\ \mathcal{V}(J)_{x_1}(\sigma) &= \sum_V P_{\mathrm{VJ}}(V,J)P_{\mathrm{delV}}(l_V-x_1|V)\mathbb{I}((\mathbf{s}^V_{1:x_1},\sigma)\sim\mathbf{a}_{1:i_1}) \quad \text{if } u_1=2,\\ \mathcal{V}(J)_{x_1} &= \sum_V P_{\mathrm{VJ}}(V,J)P_{\mathrm{delV}}(l_V-x_1|V)\mathbb{I}(\mathbf{s}^V_{1:x_1}\sim\mathbf{a}_{1:i_1}) \quad \text{if } u_1=3.\end{aligned} \tag{27}$$

2. $\mathcal{M}^{x_1}{}_{x_2}$

Contribution from the non-templated N insertions (VJ junction). $\mathcal{M}^{x_1}{}_{x_2}$ is identical to the definition of $\mathcal{M}^{x_1}{}_{x_2}$ from the VDJ algorithm (except using the parameters $S_{\mathrm{VJ}}, P_{\mathrm{insVJ}}$, and p_0 from a VJ recombination model).

3. $\mathcal{J}(J)^{x_2}$

Contribution from the templated J genes.

$$\begin{aligned}\mathcal{J}(J)^{x_2}(\tau) &= P_{\mathrm{delJ}}(d_J|J)\mathbb{I}(s^J_{d_J+1}=\tau)\mathbb{I}(\mathbf{s}^J_{d_J+1:l_J}\sim\mathbf{a}_{i_2:L}) \quad \text{if } u_2^*=1,\\ \mathcal{J}(J)^{x_2}(\tau) &= P_{\mathrm{delJ}}(d_J|J)\mathbb{I}((\tau,\mathbf{s}^J_{d_J+1:l_J})\sim\mathbf{a}_{i_2:L}) \quad \text{if } u_2^*=2,\\ \mathcal{J}(J)^{x_2} &= P_{\mathrm{delJ}}(d_J|J)\mathbb{I}(\mathbf{s}^J_{d_J+1:l_J}\sim\mathbf{a}_{i_2:L}) \quad \text{if } u_2^*=3.\end{aligned} \tag{28}$$

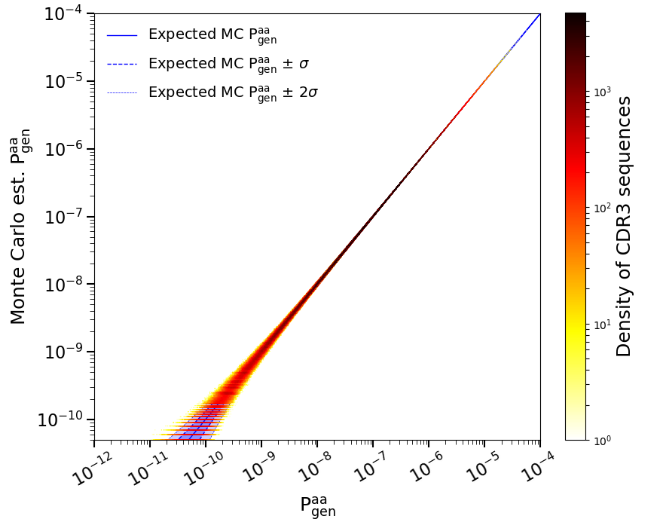

FIG. S3: Monte Carlo estimate of the generation probability of amino acid human TRA CDR3 sequences, $P_{\rm gen}^{\rm aa}$, versus OLGA's calculation. The horizontal lines at the lower left of the plot represent CDR3s that were generated once, twice, etc, in the MC sample. The one- and two-sigma curves display the deviations from exact equality between simulated and computed $P_{\rm gen}$ to be expected on the basis of Poisson statistics.

where $dJ = l_J - 3L - x - 1$

This algorithm is validated in the same manner to the VDJ algorithm, i.e. comparing to Monte Carlo (MC) estimation (Fig S3).

III. DEPENDENCE ON MODEL PARAMETERS AND STRUCTURE

In order to efficiently compute the summation in Eq. 3 the summations of the model contributions from each of the 5 segments of a CDR3 (V genomic, N1 insertions, D genomic, N2 insertions, and J genomic) are performed in a specific order (summarized in Fig S2). Specifically, we start at the left and right ends of the CDR3 read and move inwards, summing over positional indices at each step. As the D and J segments are correlated, it is useful to consider the V and N1 contributions separately from the D, N2, and J and to do the final summation over the index x_2 after the D, N2, and J components are summed over all D alleles (notice the D dependencies in Fig S2). This breakdown is useful to highlight the most computationally intensive steps: N2 insertions and the D alignment. These steps (along with the N1 insertions) require considering that the associated segment could begin and end at each allowed position. This is mathematically seen as the summation over positions and computing a matrix indexed by two indices, leading to an $O(L^2)$ complexity. The N2 insertions and D alignments are further aggravated due to model correlations between the D and J genes requiring repeating the steps for N2 insertions and D alignment for each D allele. The runtime of OLGA is thus most sensitive to the maximum number of N2 insertions and the length and number of the D alleles. The effects of varying these parameters is best illustrated by comparing runtimes for mouse TRB, human TRB, and human IGH models (Table S1). In a similar fashion, the most computationally intensive step of computing $P_{\rm gen}$ of a

TABLE S1: Model comparison

Species/Chain	max insertions	# D alleles	Average computation speed
Mouse TRB	11	2	70.4 seqs/CPU second
Human TRB	30	3	35.6 seqs/CPU second
Human IGH	60	35	2.05 seqs/CPU second

VJ model (e.g. human TRA) is the insertion step, and due to correlations between the V and J genes this is repeated for each J allele in a similar fashion as the D alleles However, as the J region of a human TRA is fairly large, many of these J genes can be excluded from alignment (if they contribute 0 probability), yielding the much faster computation rate of 184 seqs/CPU second.

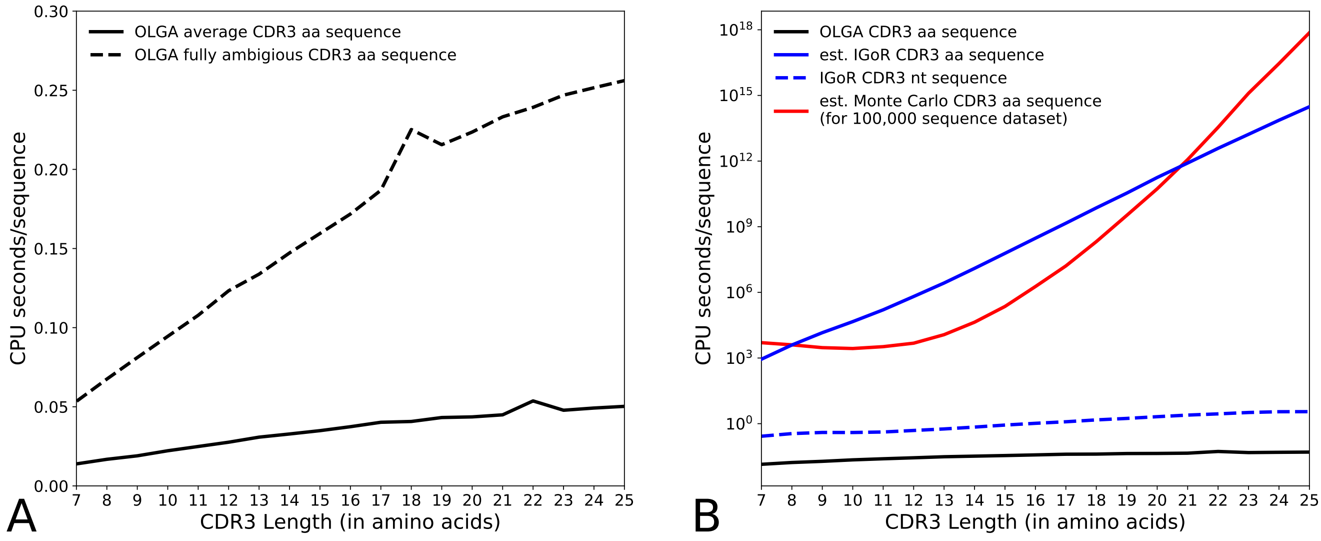

FIG. S4: A) Computational performance of OLGA as a function of CDR3 length. We compare performance averaged over a sample of human TRB amino acid CDR3 sequences to the worst case scenario of CDR3 sequences composed of fully ambiguous amino acids X. In both cases the time for a single sequence increases roughly linearly (i.e. less than the algorithmic worst case of $O(L^2)$). B) Computational performance of different P_{gen} methods as a function of CDR3 length (log scale). The IGoR and OLGA runtimes are determined by running over the same statistical sample of human TRB sequences. OLGA runs over the translated amino acid CDR3 sequences while IGoR runs over nucleotide CDR3 (dashed blue line). In order to compare OLGA to how long it would take IGoR to compute P_{gen} of amino acid CDR3s we estimate by multiplying the IGoR runtime of single nucleotide sequences (dashed blue line) by the number of nucleotide sequences that translate to the given amino acid sequence (yielding the solid blue line). Monte Carlo runtime is estimated for a dataset of 100,000 sequences with an estimated coverage of 66% of sequences having at least one count. OLGA vastly outperforms both direct enumeration (est. IGoR) and Monte Carlo.

IV. TIMING, PERFORMANCE, MODEL DEPENDENCE

In order to analyze OLGA's computational performance as a function of CDR3 length, and to compare to other hypothetical methods, we use the human TRB model as an example.

As discussed in the previous section, the most computationally intensive steps of OLGA (N1, N2, and D) require at most $O(L^2)$ operations. In practice, OLGA's scaling of the computation speed as a function of CDR3 length, even for the worst case sequences, i.e. fully ambiguous amino acids of a given length, is closer to linear in the relevant regime due to the finite parameterization of the model (maximum number of insertions, maximum size of D sequences, etc). This is shown in Fig S4A.

We also compare OLGA to runtimes of IGoR (i.e. direct enumeration of recombination events) and a hypothetical Monte Carlo computation (Fig S4). As we will explain, neither the IGoR nor the MC are precise comparisons to OLGA, yet OLGA is faster than either.

The IGoR runtimes are for nucleotide sequences not amino acid sequences. In order for IGoR to compute the P_{gen} of an amino acid sequence, it would need to compute and sum the P_{gen} of each nucleotide sequence that codes for the amino acid sequence. These sequences can be enumerated for extremely short CDR3 lengths, however the number explodes exponentially in CDR3 length. Even for a CDR3 length of 4, by enumerating all nucleotide sequences for an amino acid sequence IGoR computes 0.33 seqs/CPU second compared to the 122 seqs/CPU second for OLGA. For longer CDR3 lengths we approximate how long IGoR would take by computing the average number of CDR3 nucleotide sequences per CDR3 amino acid sequence for a given length. OLGA not only heavily outperforms this exponential blowup, but actually outperforms IGoR when IGoR is computing a *single* nucleotide sequence of a given amino acid sequence.

The Monte Carlo runtime estimate comes from the setup of estimating the P_{gen} of 100,000 sequences. These P_{gen} would be estimated by simulating enough recombination events such that 66% of CDR3 sequences of a given length would be expected to have at least one count. There is a CDR3 length scaling due to the trend that shorter sequences tend to have higher P_{gen} (Fig S5B). The P_{gen} estimated using this methodology will be extremely noisy (Poisson noise on the expected number of counts) and not even give reliable estimates for many sequences.

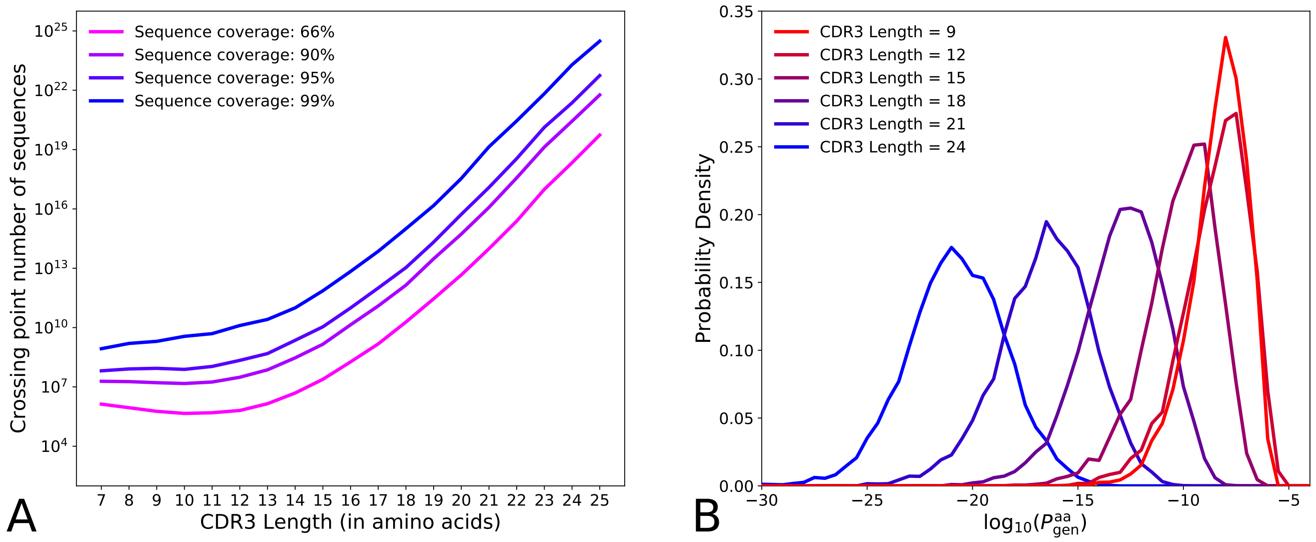

FIG. S5: A) The runtime of Mont Carlo P_{gen} estimation scales as $1/P_{\text{gen}}$ while OLGA will scale with the number of sequences. This predicts a number of sequences for the 'crossing point' where the runtime of Monte Carlo P_{gen} estimation is comparable to OLGA P_{gen} for sequences with P_{gen} above some cutoff. For datasets with more sequences than these curves, Monte Carlo estimation may be faster (depending on the level of Poisson noise considered tolerable), while below these curves OLGA is always faster. We plot this as a function of CDR3 length where the P_{gen} cutoffs are determined to ensure that on average some fraction (66%, 90%, 95%, and 99%) of the sequences at that length get covered by the Monte Carlo estimation. B) $\log_{10}(P_{\text{gen}})$ probability density distributions for a few examples of CDR3 lengths. These curves are used to determine the MC P_{gen} cutoffs per CDR3 length by determining, for a given curve, when the area under the curve and right of a P_{gen} cutoff matches the sequence coverage fraction.

It is true that the computation time for MC estimates scale as $1/P_{\text{gen}}$ and not with the number of sequences. Thus, there is a hypothetical number of sequences when MC is faster than OLGA if we are willing to accept noisy estimates and to entirely miss some fraction of the CDR3s. This 'crossing point' number of sequences is plotted in Fig S5A and corresponds to completely unrealistic numbers of sequences, highlighting the fact that OLGA will not only give a more reliable P_{gen}, even for very unlikely sequences, but is also much faster than MC even for short, high P_{gen}, sequences. So, even overlooking the drawbacks and imprecision of MC estimation, for plausible sized datasets OLGA is still dramatically faster than MC.

V. GENERATION PROBABILITY DISTRIBUTIONS FROM RNA-DERIVED REPERTOIRES

The analyses described in the main text were mostly concerned with datasets derived by sequencing the genomic DNA contained in a sample of immune cells to directly obtain sequences of the rearranged TCR genes. Immune repertoires can alternatively be obtained by sequencing the mRNA expressed from the same genes, and many such RNA-based data sets exist. Given a TCR sequence, OLGA evaluates the probability of the primitive recombination event (or events) that must have occurred to create the initial T cell carrying that sequence, and the applicability of OLGA is independent of how the sequence was obtained (i.e. from DNA or RNA sequencing). OLGA relies on the availability of a suitable recombination model but that model is thought to vary very little with time (and disease status) for each individual subject and only moderately from individual to individual in a given species. The probability that a given sequence, once generated in a primitive event, will be captured in a sequencing experiment is at best roughly constant across sequences, and may vary substantially between different capture protocols.

For these reasons, it is interesting to investigate how these generation probability distributions vary across CDR3 repertoires obtained using different sequencing protocols in different biological contexts. In Fig. S6 we plot the results of running OLGA on a few recently published human TRB repertoires that were obtained using RNA sequencing. These samples comprise a study of patients with glioblastoma disease (Sims *et al.* [4]), a study of patients with Crohn's disease and ulcerative colitis (Wu *et al.* [6]), and a comprehensive study of the dynamics of TCRs in healthy individuals (Wang *et al.* [5]). Fig. S6 shows the generation probability distribution of data sets from these three sources, for comparison plotted together with the distribution obtained from DNA sequencing of the large human

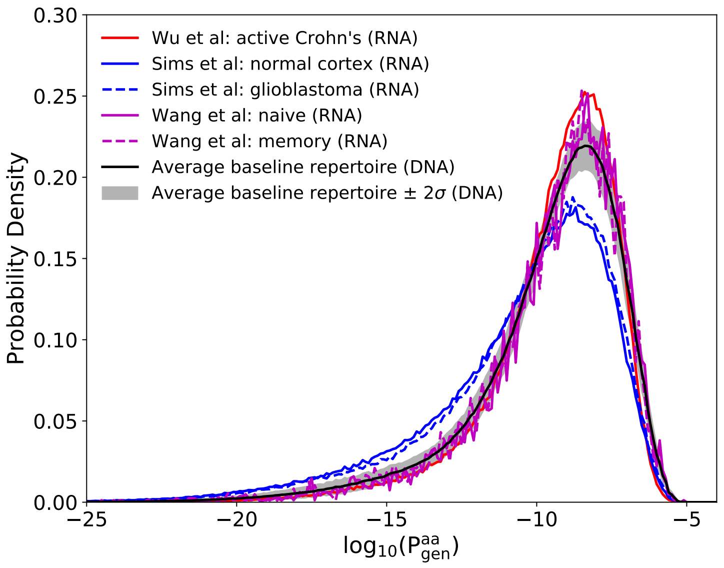

FIG. S6: Generation probability distributions for TRB CDR3 sequences taken from three different sources (Sims *et al.* [4], Wu *et al.* [6], Wang *et al.* [5]), compared to DNA RepSeq data from Emerson *et al.* [2] (black curve with standard deviation), using a model inferred from [2]. All distributions have approximately the same shape, with a slight bias in the data from Sims *et al.* [4], indicating how robust is the distribution. Data from Sims *et al.* are identified in the SI of [4] as IDs N01 (normal cortex) and G10 (glioblastoma). Data from Wang *et al.* is identified by Short Read Archive (SRA) accession numbers SRR030702 (naive) and SRS007450 (memory). See also Fig. S7.

sample of Emerson *et al.* [2]. As can be seen, two of the three RNA data sets give results quite consistent with the DNA-based results. The glioblastoma data (Sims *et al.* [4]) gives a distribution broadly similar to the other three, but with a systematic shift to higher frequency of occurrence of lower generation probability sequences. We do not know whether or not this difference is biologically significant, or an artifact of the used protocol. The difference does not seem to be due to sampling depth, as can be seen in Fig. S7, where multiple samples from Sims *et al.* [4] are plotted: the distributions derived from smaller samples are noisier than, but statistically consistent with, the distributions based on the largest samples.

VI. CROSS-SPECIES $P_{\rm gen}$

TCR sequence repertoires are different in detail between species, both because the genomic templates differ and because of differences in the parameters of the recombination process itself. As a result, there are clear interspecies differences in CDR3 length distribution and amino acid composition. Nevertheless, the TRB CDR3 regions of different vertebrate species have the same overall structure and the same conserved residues at the two ends of the CDR3. As a result, a CDR3 from one species usually has a non-zero probability to be produced within a different species, a fact of some interest in the context of studies of cross-species sharing of T cell types. We explored this concept with OLGA by feeding TRB CDR3s produced by the human generation model to a mouse generation model and vice versa. The resulting generation probability distributions are plotted in Fig. S8.

The sequences that are produced in one species have substantially lower probability of being generated in the other species (Fig. S8). The effect is strongest for finding human sequences in a mouse repertoire (compare the black dashed curve with red solid curve): the bulk of the human sequences have extremely low generation probabilities in the mouse model. The effect is less strong for finding mouse sequences in a human repertoire (compare the red dashed curve with the black solid curve): a small fraction of the mouse sequences have generation probabilities that are as high as the highest generation probabilities of human sequences. The results are not symmetric - while the mouse TRBs processed using the human model have a distinct bi-model distribution, the human TRBs have a very flat and low mouse generation probabilities. Furthermore, mouse TRB sequences always have a non-zero probability of being generated in a human TRB context, however 27.4% of human TRB sequences have $P_{\rm gen} = 0$ as defined by a mouse TRB model. This asymmetry is primarily due to differences in the insertion profiles (humans may have many more

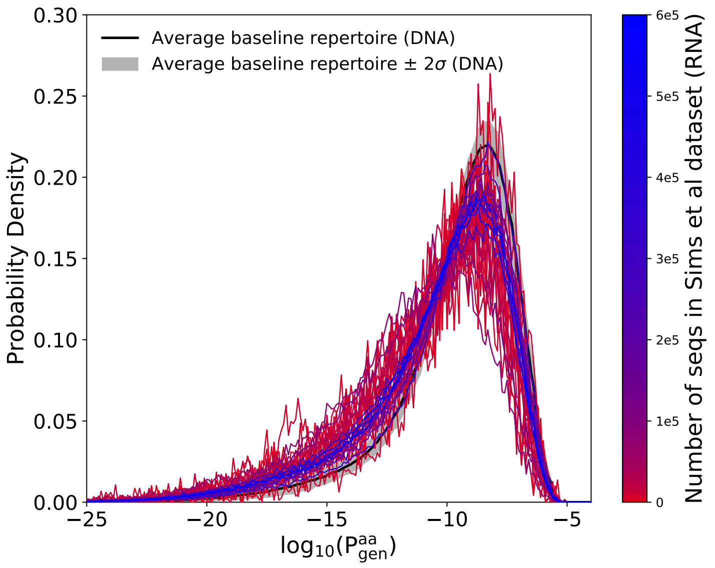

FIG. S7: Generation probability distributions for different samples from Sims *et al.* [4], compared to DNA RepSeq data from Emerson *et al.* [2] (black curve with standard deviation), using a model inferred from [2]. Color indicate sample size: larger datasets are blue, while small ones are red. The only effect of decreasing the sample size is increasing the noise, but the shape stays the same. All TRB datasets from the study are plotted.

inserted N1 and N2 nucleotides) and by extension CDR3 length. Nonetheless, these results suggest that there will be a non-negligible amount of sharing, entirely due to chance statistics, of CDR3 sequences between mouse and human repertoires. A more detailed view of this structure can be seen in a scatter plot of the generation probabilities between the two (Fig. S9). While there are many sequences with high generation probabilities in both the actual generative model and the cross species model, the cross species generation probabilities are much more variable and span many orders of magnitude, without much correlation to the correct species model.

VII. GENERATION PROBABILITY DISTRIBUTIONS FROM ADDITIONAL PATHOGEN RESPONSE DATASETS

In the main text, we displayed the distribution of generative probabilities for T cells known to respond to various pathogens, and even specific epitopes of particular pathogens. The T cell sequences are taken from databases that compile results from multiple experiments. We found that these distributions were, within statistical noise, indistinguishable from the background $P_{\rm gen}$ distribution of PBMCs drawn from the blood. In other words, it would seem that there is no correlation between ease of generation of a T cell and its likelihood to respond to a particular pathogen or epitope. A defect of this analysis is that the database agglomerates sequences from different experimental protocols, so that there is no way of knowing what biases might have affected the inclusion of any given sequence in the database. Obviously, it would be better to do a single well-controlled experiment in which T cells from a single donor are stimulated to expand by selected pathogens, and the expanded T cells sequenced. Such an experiment was reported by Becattini *et al.* [1] several years ago. In their experiment, CD4+ helper T cells were separated from peripheral blood samples, autologous monocytes from the same samples were incubated with three different pathogens (a fungus, a bacterium, and a toxin) in order to load pathogen epitopes, and helper T cell subsamples (typically containing several million T cells, and hundreds of thousands of clonotypes) were incubated with the prepared monocytes (this was done independently for samples from several donors). The T cells in the various samples that had proliferated under this treatment were separated out (typically yielding millions of cells) and their TRB sequences obtained using the Adaptive Biotechnology genomic DNA protocol. The result is a collection of lists of clones (defined by CDR3 amino acid sequence) from the blood of individual donors that can be said to have expanded under stimulation by the three different pathogens. The responses obtained in this way are quite polyclonal, with a few thousand clonotypes in each list of responding clones (the polyclonality perhaps being due to the fact that stimulation is with preparations of whole pathogens, as opposed to particular pathogen peptides). The $P_{\rm gen}$ distributions of the pathogen–responsive

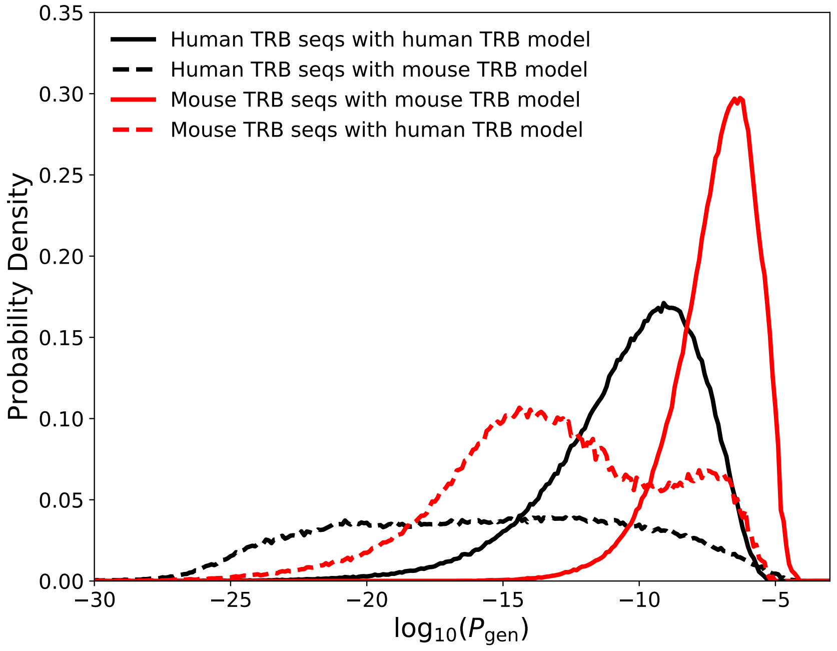

FIG. S8: Probability densities of $\log_{10}(P_{\rm gen})$ for sequences generated from mouse TRB and human TRB models. The $P_{\rm gen}$ of a sequence is computed using either a mouse TRB model or a human TRB model depending on the curve. Models are based on data from Emerson *et al.* [2] for human TRB and Sethna *et al.* [3] for mouse TRB.

clones for different individuals and pathogens are plotted in Fig. S10. They are indistinguishable from the background $P_{\rm gen}$ distribution derived from blood samples of healthy individuals, which is also plotted (along with its two-sigma variance across a population of individuals) for reference. These data further strengthen the conclusion that pathogen response activity is uncorrelated with $P_{\rm gen}$.

---

[1] Becattini, S., Latorre, D., Mele, F., Foglierini, M., De Gregorio, C., Cassotta, A., Fernandez, B., Kelderman, S., Schumacher, T. N., Corti, D., Lanzavecchia, A., and Sallusto, F. (2015). Functional heterogeneity of human memory cd4+ t cell clones primed by pathogens or vaccines. *Science*, **347**(6220), 400–406.

[2] Emerson, R. O., DeWitt, W. S., Vignali, M., Gravley, J., Hu, J. K., Osborne, E. J., Desmarais, C., Klinger, M., Carlson, C. S., Hansen, J. A., Rieder, M., and Robins, H. S. (2017). Immunosequencing identifies signatures of cytomegalovirus exposure history and HLA-mediated effects on the T cell repertoire. *Nature Genetics*, **49**(5), 659–665.

[3] Sethna, Z., Elhanati, Y., Dudgeon, C. S., Callan, C. G., Levine, A. J., Mora, T., and Walczak, A. M. (2017). Insights into immune system development and function from mouse T-cell repertoires. *Proceedings of the National Academy of Sciences*, **114**(9), 2253–2258.

[4] Sims, J. S., Grinshpun, B., Feng, Y., Ung, T. H., Neira, J. A., Samanamud, J. L., Canoll, P., Shen, Y., Sims, P. A., and Bruce, J. N. (2016). Diversity and divergence of the glioma-infiltrating t-cell receptor repertoire. *Proceedings of the National Academy of Sciences*, **113**(25), E3529–E3537.

[5] Wang, C., Sanders, C. M., Yang, Q., Schroeder, H. W., Wang, E., Babrzadeh, F., Gharizadeh, B., Myers, R. M., Hudson, J. R., Davis, R. W., and Han, J. (2010). High throughput sequencing reveals a complex pattern of dynamic interrelationships among human T cell subsets. *Proceedings of the National Academy of Sciences of the United States of America*, **107**(4), 1518–23.

[6] Wu, J., Pendegraft, A. H., Byrne-Steele, M., Yang, Q., Wang, C., Pan, W., Lucious, T., Seay, T., Cui, X., Elson, C. O., Han, J., and Mannon, P. J. (2018). Expanded tcrcdr3 clonotypes distinguish crohn's disease and ulcerative colitis patients. *Mucosal Immunology*, **11**(5), 1487–1495.

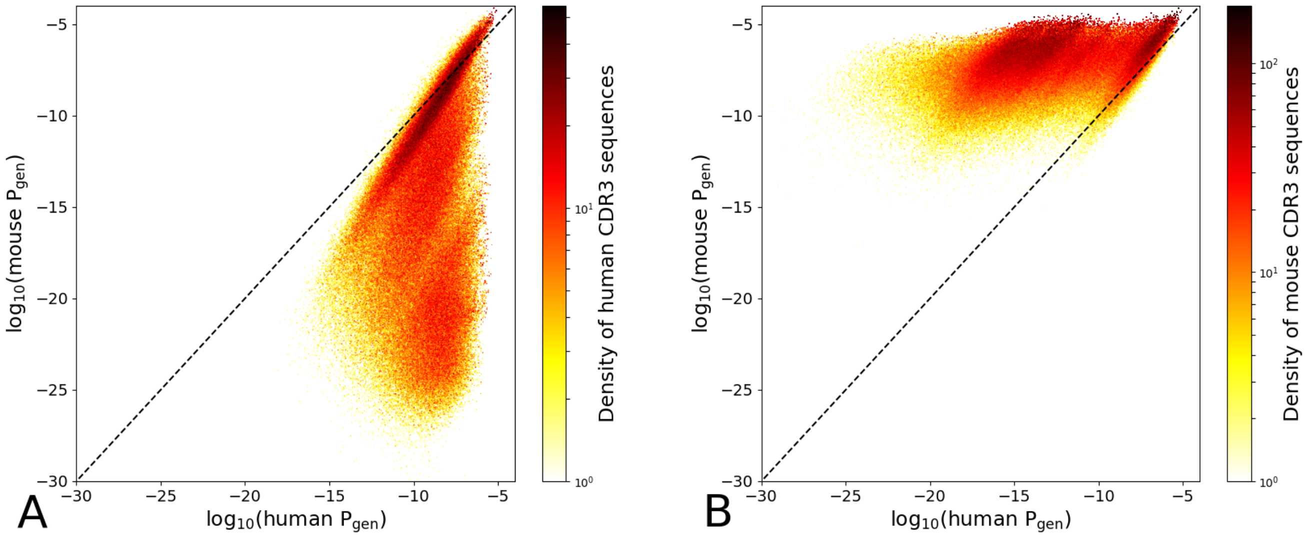

FIG. S9: Scatter plots of CDR3 sequence repertoires across their P_{gen} values as determined by a human TRB model or a mouse TRB model. The sequence repertoires are Monte Carlo samples from A) a human TRB model or B) a mouse TRB model. Projections of the scatter plots onto the two axes reproduce the distributions displayed in Fig. S8. Models are based on data from Emerson *et al.* [2] for human TRB and Sethna *et al.* [3] for mouse TRB.

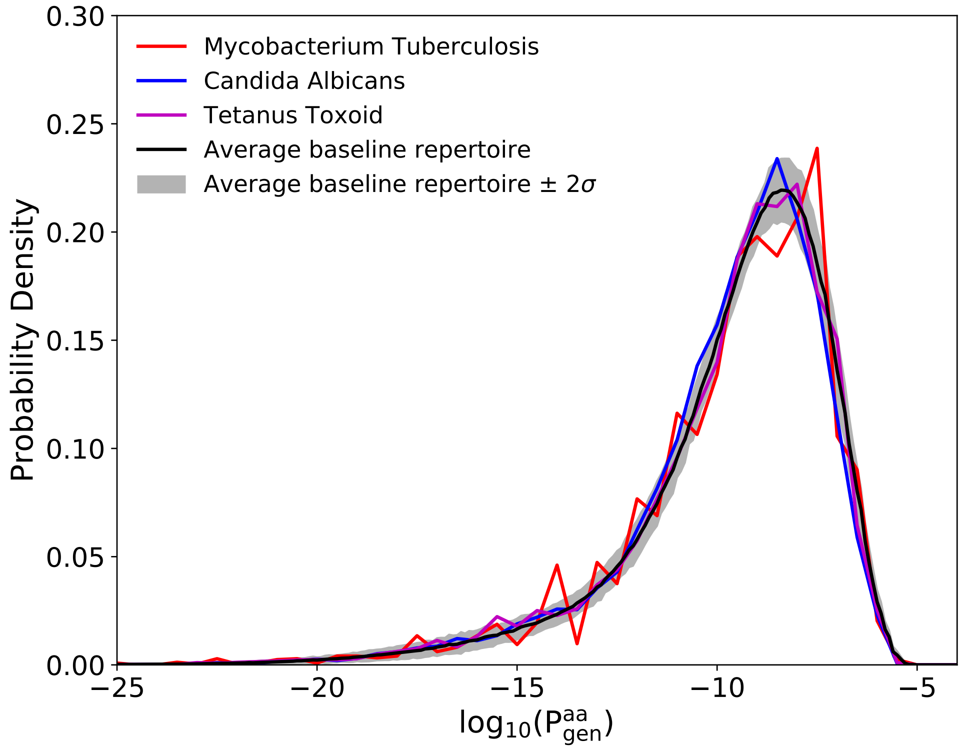

FIG. S10: P_{gen} distributions for human CD4+ T cell repertoires that have been incubated with three different pathogens (Becattini *et al.* [1]): the fungus Candida Albicans (CA), the bacterium Mycobacterium Tuberculosis (MT), and a toxin protein Tetanus Toxoid (TT). For comparison, the background distribution from human peripheral blood TRB sequences from Emerson *et al.* [2] (with its two sigma variation across multiple individuals) is also plotted. The plotted curves are averages over data from individual donors. The sizes of the responsive T cell repertoires are quite variable: the CA dataset has 39934 clonotypes from 5 donors, the TT dataset has 26573 clonotypes from 4 donors, and the MT dataset has 5082 clonotypes from 2 donors. The generation model was infered from Emerson *et al.* [2].
